# Increasing staff confidence about domestic abuse identification, disclosure and safeguarding in a community mental health team

**DOI:** 10.1192/bjo.2021.413

**Published:** 2021-06-18

**Authors:** Beth McCausland, Nicola Minicozzi, Siobhan O'Halloran, Avril Ward, Kerry Elliott

**Affiliations:** 1 Southern Health NHS Foundation Trust, University of Southampton; 2 Yellow Door, Pathfinder; 3 Aurora New Dawn Ltd, Pathfinder

## Abstract

**Aims:**

To increase staff confidence about identifying Domestic Abuse (DA), particularly regarding ‘how to ask’ to encourage disclosure and the pathways available for appropriately safeguarding survivors; in a Community Mental Health Team (CMHT) setting.

**Background:**

DA is bi-directionally associated with mental health (MH) disorders; 1:4 women in contact with MH services are currently experiencing DA. MH professionals (MHPs) are in a privileged position to identify DA and support survivors. However, this is dependent on MHPs receiving adequate training about DA. For this, we collaborated with Pathfinder, a national pilot project run by a consortium of five expert partners that aims to establish comprehensive health practice in relation to DA and Violence Against Women & Girls in Acute Hospital Trusts, MH Trusts and Primary Care. In Southampton, Pathfinder has funded two domestic and sexual abuse (DSA) advocates to both train MH staff and take a small caseload of MH service users who are experiencing abuse.

**Method:**

We conducted a baseline survey of staff confidence across the following domains:

Knowing the legal definition of DA,

The process used to escalate a DA concern,

How to make a referral,

How to complete DASH forms,

How and when to refer to Pathfinder,

What the following acronyms mean: PIPPA, MAPPA, MARAC, IDVA, DASH,

What HRDA and MASH mean,

How to ask about DA,

Who to signpost service users to if they make a disclosure, and when to involve the police.

We presented the survey results at the regional Pathfinder strategic group, with Trust management representatives present. This project fits within the strategic group's sustainability aims to increase DA awareness and safeguarding processes across the Trust.

The Pathfinder funded DSA Advisors delivered a four-hour training package targeting the surveyed questions and wider information on DA. We then re-surveyed to see if staff confidence had increased. We are currently analyzing the number of referrals to the Pathfinder service pre- and post-training.

**Result:**

Staff confidence increased across all domains following the training (% mean increase): Qs1 (35%), Qs2 (9%), Qs3 (45%), Qs4 (81%), Qs5 (25%), Qs6 (49%), Qs7 (89%), Qs8 (62%) and Qs9 (48%).

We have now arranged a bi-monthly drop-in at the CMHT by the DSA advisor who provided the training, to embed the link between the services and maintain staff confidence. We will circulate these results to advocate that this training is provided across the Trust.

